# Two-Step Aging of Highly Polar Glass

**DOI:** 10.1021/acs.jpclett.1c03572

**Published:** 2021-12-02

**Authors:** Zaneta Wojnarowska, Marian Paluch

**Affiliations:** August Chełkowski Institute of Physics, Silesian Center for Education and Interdisciplinary Research, University of Silesia in Katowice, 75 Pulku Piechoty 1a, 41-500 Chorzow, Poland

## Abstract

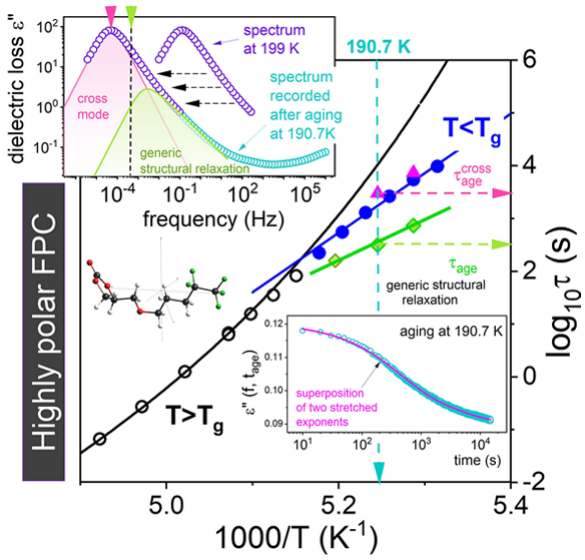

Nonequilibrium processes, including physical aging, belong to the most challenging phenomena of glassy dynamics. One of the fundamental problems that needs clarification is the effect of material polarity on the time scale of the structural recovery of glass. The importance of this issue arises from practical applications and recent findings suggesting a substantial contribution of dipole–dipole interactions to the dielectric permittivity spectra of polar glass-formers. Herein, we use dielectric spectroscopy to investigate structural relaxation and aging dynamics of highly polar glass-former 4-[(4,4,5,5,5-pentafluoropentoxy)methyl]-1,3-dioxolan-2-one (FPC), a derivative of propylene carbonate with ε_s_ = 180 and μ = 5.1. We show that ε″(*t*_age_) data of FPC at *T*_age_ < *T*_g_ reveal complex behavior resulting from considerable cross-correlation effects. Namely, two characteristic aging time scales, reflecting the evolution of cross-correlation mode and generic structural relaxation toward equilibrium, are obtained at a given *T*_age_. Furthermore, a single stretched exponential behavior of ε″(*t*_age_) has been received for weakly polar carvedilol with negligible dipole–dipole interactions.

Noncrystalline solids (glasses) play an essential role in practically all fields of material science.^1^ In contrast to the supercooled state, the amorphous phase reveals the nonequilibrium nature, and therefore, its thermodynamic and dynamic properties evolve over time.^2^ For instance, the specific volume, enthalpy, relaxation dynamics, or dc-conductivity change during the equilibration process below the glass transition temperature, *T*_g_.^3^ This phenomenon, known as structural recovery or physical aging, can ultimately affect the use of diverse materials, including polymers,^4^ ion-conductors,^5^ inorganic glasses,^6^ composites, or pharmaceuticals,^7^ in modern technologies. Therefore, it is essential to monitor the fluctuations of physical quantities after quenching. Experimentally, this can be realized by many techniques; however, only one, dielectric spectroscopy (DS), can follow the aging process precisely over an extraordinary range of external parameters, *e.g.*, temperature, pressure, or electric field.

In typical dielectric measurement, material polarization (*P*), an effect of the sinusoidal electric field (*E*), is monitored and recalculated to frequency-dependent complex permittivity ε*(ω) = (P*(ω) + ε_0_E*(ω))/ε_0_E*(ω) = ε′(ω) – iε″(ω).^8^ When the experiment is performed in equilibrium supercooled state (at *T* > *T*_g_), a frequency sweep delivers the structural (α) relaxation peak of the imaginary part of dielectric permittivity ε″(ω) and step-like change observed at the same time on ε′(ω).^9^ Such data are then modeled by the Havriliak–Negami expression, ε*(ω) = ε_∞_ +  or Kohlrausch–Williams–Watts (KWW) function, ϕ(*t*) ∼ exp[−(*t*/τ)^β_KWW_^] to characterize the structural dynamics quantitatively.^10^ Importantly, significant differences are observed when comparing the dielectric permittivity spectra ε″(ω) of various glass formers with respect to the primary relaxation process. Namely, the stretching parameter β_KWW_ takes the values 0.5–0.8 depending on system polarity. Recently, a universal correlation between the width of α-process and the dielectric relaxation strength *Δε* (reflecting the magnitude of dipole moment) has been found for 88 van der Waals systems.^11^ Specifically, the more polar the glass-former, the larger the Δε and the narrower the α-loss peak. This finding highlighted the sensitivity of dielectric spectroscopy toward so-called cross-correlations between dipoles existing in polar systems. In contrast, other experimental methods that monitor the structural dynamics, *e.g.*, depolarized dynamic light scattering (DDLS) or mechanical spectroscopy, show the universal spectral shape of the α-process for polar and weakly polar compounds.^12^ In particular, a high-frequency power-law of ω^–1/2^ is generally observed.^13^ Notably, DS recovers such a generic behavior only for systems with a low dipole moment. Hence, recently it has been postulated that additional contributions originating from cross-correlations between dipoles mask the structural (generic) relaxation in ε″(ω) spectra of strongly polar liquids. Experimental results supporting this hypothesis have been presented recently for monohydroxy alcohols, glycerol and TBP.^13,14^ In particular, two processes have been distinguished in the dielectric response of these compounds: (i) a slower Debye-like process identified with cross-correlations (being due to H-bonded supramolecular structures in monohydroxy alcohols) and (ii) a faster contribution with a high-frequency power law of −0.5 corresponding to the generic structural relaxation and revealing the same characteristics as relaxation processes monitored by a DDLS technique.^15^

From this viewpoint, a fundamental question arises: *Do the diverse contributions to the permittivity spectrum ε*″*(ω) display different dynamics regarding structural aging?* Herein, to address this problem, we have chosen 4-[(4,4,5,5,5-pentafluoropentoxy)methyl]-1,3-dioxolan-2-one, a room-temperature liquid supplied from SynQuest Lab (United States) and abbreviated as FPC. As a derivative of propylene carbonate (PC, μ = 4.9), FPC reveals a high dipole moment and thus a significant contribution of cross-correlations to the dielectric loss data. To approach the problem comprehensively, we have also selected carvedilol (CRV) with dielectric strength *Δε* = 2 and β_KWW_ = 0.54, proving the weak polarity of the CRV molecule and negligible cross-correlation effects. The results of our dielectric aging experiments demonstrate that structural relaxation and recovery time scales are not the same, *i.e.*, τ_age_ ≠ τ_α_ for both studied compounds, which follows the suggestions made in the recent Perspective by Richert *et al*.^16^ Furthermore, we show that structural recovery dynamics becomes more complex when cross-correlation effects dominate in polar systems. In particular, we obtained two characteristic aging time scales corresponding to cross-mode and generic structural relaxation.

According to our differential scanning calorimetry (DSC) measurements, FPC with *T*_g_ = 197.2 K and no crystallization tendency can be classified as an excellent glass-forming liquid. Consequently, it is more advantageous for aging experiments than PC, which forms a crystalline phase spontaneously.^17^ To provide an insight into the relaxation dynamics of FPC, the temperature-dependent dielectric measurements were carried out with a Novocontrol Alpha analyzer connected to a Novocool controller. The spectra were collected on cooling with the use of a capacitor described in ref (18). The obtained real and imaginary parts of complex dielectric permittivity ε*(*f*) are presented in panel A and B of Figure 1, respectively.

**Figure 1:**
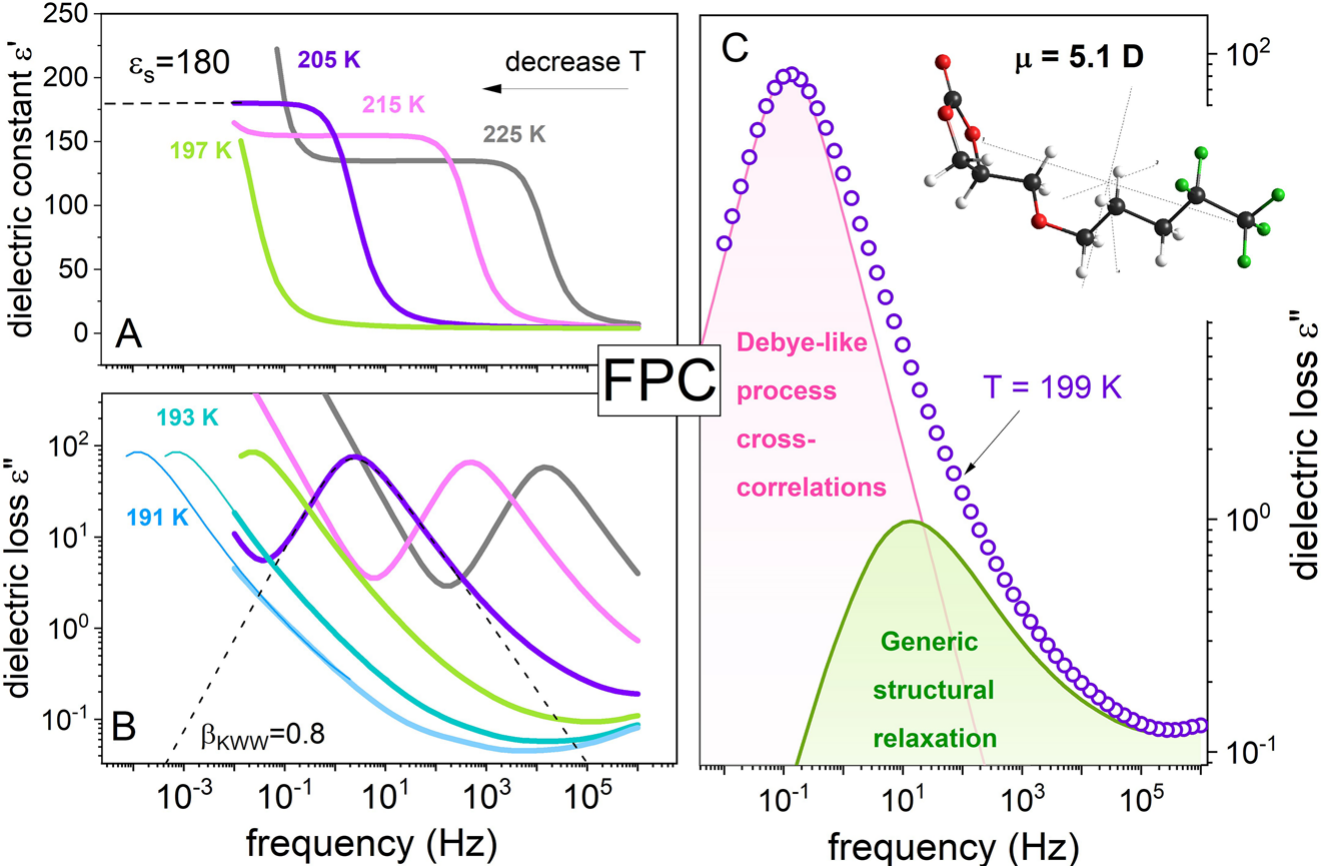
Real ε′(*f*) (A) and imaginary ε″(*f*) (B) part of dielectric permittivity of FPC at several representative temperatures. Each data curve at *T* below 197 K was measured after fast cooling from *T*_eq_ = 205 K. The dashed line denotes the fit of the KWW function with β_KWW_ = 0.8. Panel C presents the dielectric loss data of FPC recorded at 199 K. The cross-contribution and generic structural relaxation to the dielectric spectra have been obtained by fitting experimental data with the Debye and HN function superposition. The inset presents the chemical structure of FPC (red, oxygen; green, fluorine; black, carbon; gray, hydrogen).

As can be seen, FPC follows the behavior typical for glass-forming liquids,^19^*i.e.*, the dielectric loss peak ε″(*f*) moves toward lower frequencies with cooling, and the static dielectric constant ε_s_ is getting higher at the same time. However, the value of ε_s_ = 180 in the vicinity of the liquid–glass transition is much higher than that found for canonical glass-former propylene carbonate (ε_s_ = 102),^20^ which is consistent with a significant dipole moment of FPC molecule (μ = 5.1 determined in *ab initio* calculations). In turn, from Figure 1C it can be easily noticed that the dielectric loss curve of FPC, recorded in close vicinity of *T*_g_, is only slightly broader than the Debye process (β_KWW_ of FPC is equal to 0.80). Thus, FPC belongs to liquids of the narrowest distribution of relaxation times. In this context, a substantial role of dipole–dipole interactions to the dielectric spectra ε″(*f*) is expected for FPC. This is schematically presented in Figure 1C.

In a further step of our study, the isothermal glass equilibration at three different temperatures, in close vicinity of *T*_g_, has been performed for FPC. According to the typical protocol, the aging experiment contains two steps: (i) temperature jump from an initial equilibrium (*T*_eq_ > *T*_g_) to nonequilibrium state (*T*_age_ < *T*_g_) at a time *t* = 0 (herein, *T*_eq_ was chosen as 205 K) and (ii) measurements of dielectric response on a time scale shorter than the aging process itself.^21^ Taking into account the later requirement, the dielectric loss at fixed frequency ε″(*f̃*) has been monitored over time. Additionally, the dielectric curve in a broad frequency range was measured as a final point. When the glass is densified with an aging time *t*_age_, ε″ at a given frequency continuously decreases, reflecting the evolution of structural changes.^4,22^ The same has been observed for FPC. To directly compare the structural recovery time scale at various temperature conditions, the ε″(*f̃*, *t*_age_) data of FPC have been normalized by initial ε″_*t*→0_ and final plateau ε″_*t*→∞_ value of the loss permittivity. As presented in Figure 2A, the exponential decay of ε″(*f̃*, *t*_age_) was obtained at each examined temperature; however, the closer the liquid–glass transition, the faster the evolution of ε″ toward equilibrium is observed.

**Figure 2:**
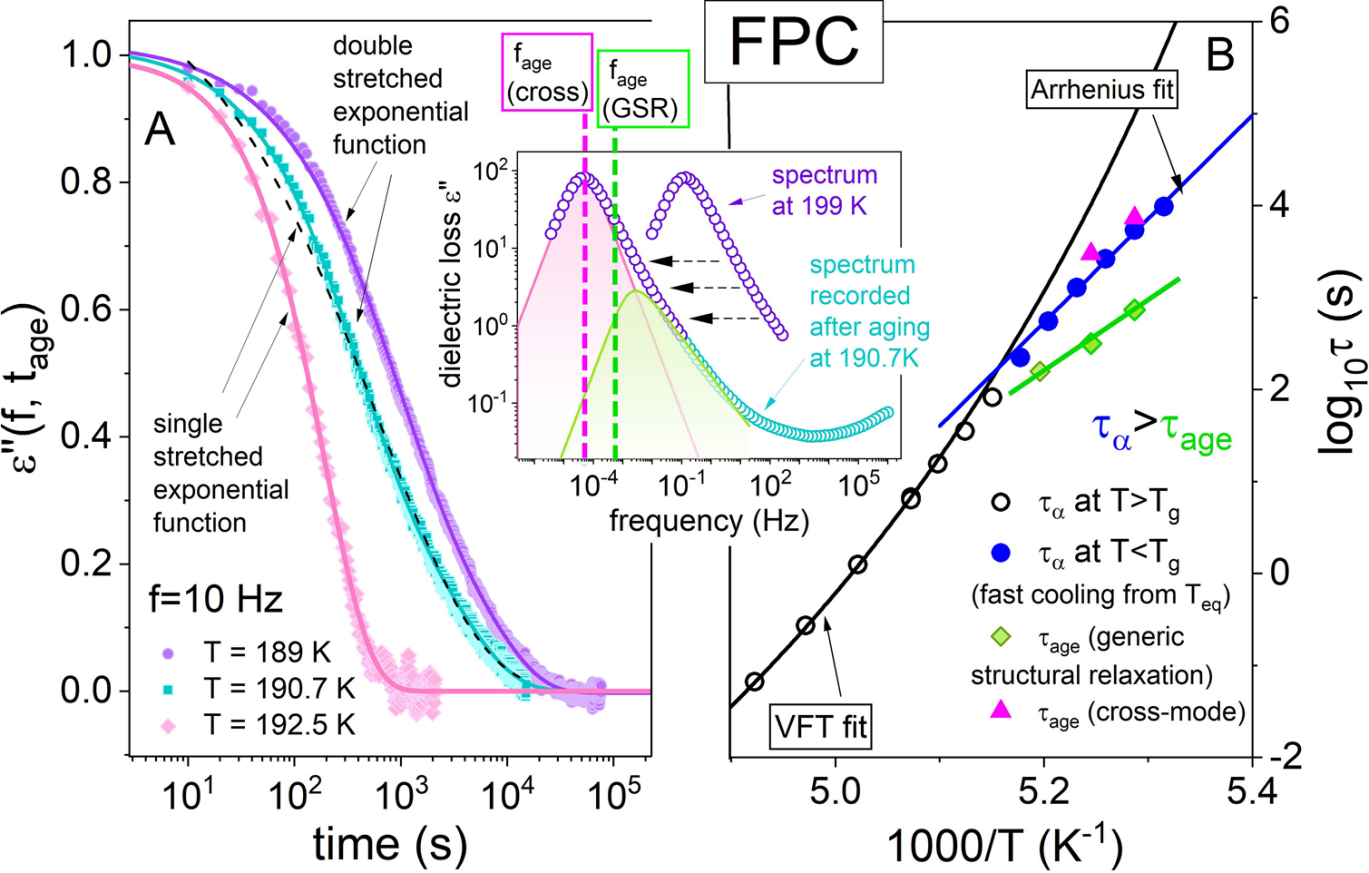
Normalized kinetic curves recorded at three temperatures during the dielectric aging experiment (A). Normalization has been made by using the following equation (ε″(t) – ε″_t→∞_)/(ε″_t→0_ – ε″_t→∞_). A solid pink line is a fit of a single stretched exponential function (eq 1) to the experimental data with parameters τ_age_ = 190 s and β_ag_ = 1. The data recorded at 190.7 and 189 K have been fitted with eq 2 and τ_age1_ = 333.6 s, β_ag1_ = 0.76, τ_age2_ = 2569 s, β_ag2_ = 0.58 for the former and τ_age1_ = 777.6 s, β_ag1_ = 0.66, τ_age2_ = 8518 s, and β_ag2_ = 1 for the latter. The inset presents the dielectric loss curve recorded at the equilibrium achieved at 190.7 K. The data have been extended to the low-frequency range by a horizontal shift of spectrum recorded at 199 K. Dashed lines visualize the frequencies corresponding to τ_age1_ and τ_age2_ determined as fit parameters at this *T*. Panel B presents the temperature dependence of structural relaxation times of FPC determined above and below *T*_g_ together with the results from aging experiments. The data obtained at *T* < *T*_g_ and those from the aging experiment were obtained after a temperature jump from *T*_eq_ = 205 K.

To quantify the change in dielectric permittivity accompanying the aging process, initially, we have parametrized the kinetic curves by using the stretched exponential (KWW) function in the form
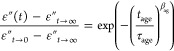
1where τ_age_ means the aging time constant and β_ag_ is the stretching exponent. However, a satisfactory fit has been obtained only for equilibration taking place at 192.5 K, *i.e.*, 5 K below *T*_g_^DSC^ (see the solid pink line in Figure 2A). A clear deviation of the fitting curve from experimental points has been observed in two other cases (see the dashed black line in Figure 2A as an example). Therefore, we used the superposition of two KWW functions to parametrize the data precisely.
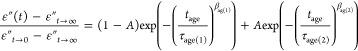
2

Consequently, two decay times τ_age_ have been obtained for kinetic curves registered at *T* = 190.7 K and *T* = 189 K. The fitting parameters are listed in the figure caption. Additionally, the values of τ_age_ are visualized in Figure 2B (solid diamonds and triangles) together with the temperature evolution of structural relaxation times τ_α_, above and below *T*_g_. The temperature dependence of τ_α_ at *T* > *T*_g_, determined directly from maxima of ε″(*f*) peaks, can be described using the VFT expression log τ = log τ_0_ + 0.434*DT*_0_/*T* – *T*_0_ with log τ_0_ = −12.5 ± 0.7 s, *D* = 6.28 ± 0.92, and *T*_0_ = 163 ± 1 K, yielding a fragility index of *m* = 91 for FPC. To extract τ_α_ below the *T*_g_, the α-peak recorded just above *T*_g_ has been shifted horizontally to the temperatures *T* < *T*_g_ so that its high-frequency side superimposes with the spectra collected in the glassy state. This operation could be employed because the shape of the structural relaxation mode does not change with *T*, *i.e.*, the time–temperature superposition (TTS) rule^23^ holds for FPC. The same procedure was also used to determine the position of α-relaxation after the equilibration at *T*_age_ (see inset to Figure 2). From the comparison presented in Figure 2B, it can be noted that there are substantial differences between two time constants τ_age_ determined at given *T*_age_; they are respectively faster and shorter than the time scale of structural relaxation in the glassy state. This implies that fast and slow relaxing modes shift to their new positions at a different rate. Specifically, fast modes get aged faster than slow ones. This result can be rationalized in the context of cross-correlation contributions to the dielectric loss spectrum. From the inset to Figure 2, one can recognize that the slower structural recovery time τ_age_ corresponds well with the maximum of α-process (or cross-correlation mode). At the same time, the second one, around one decade faster, appears on the low-frequency flank of generic structural relaxation. Note that structural generic relaxation and cross-contributions to dielectric spectrum have been estimated by fitting of ε″(*f*) data by superposition of two functions, *i.e.*, Debye-like and HN. Herein, one can also note that the ε″(*f̃*, *t*_age_) data can be satisfactorily parametrized with the value of β_ag_ = 1 for cross-mode.

If the presented explanation comes true, the vanishing of cross-correlations in weakly polar materials should lead to a single value of τ_age_ reflecting the aging process of generic structural relaxation. We have performed dielectric aging experiments of CRV to verify this hypothesis. The CRV molecule, classified as an active pharmaceutical ingredient (API), is characterized by a dipole moment μ equal to 1.9, the dielectric strength of *Δε* = 2.5, and broad distribution of relaxation times quantified by β_KWW_ = 0.54 (see Figure 3A), all confirming its weak polarity.

**Figure 3:**
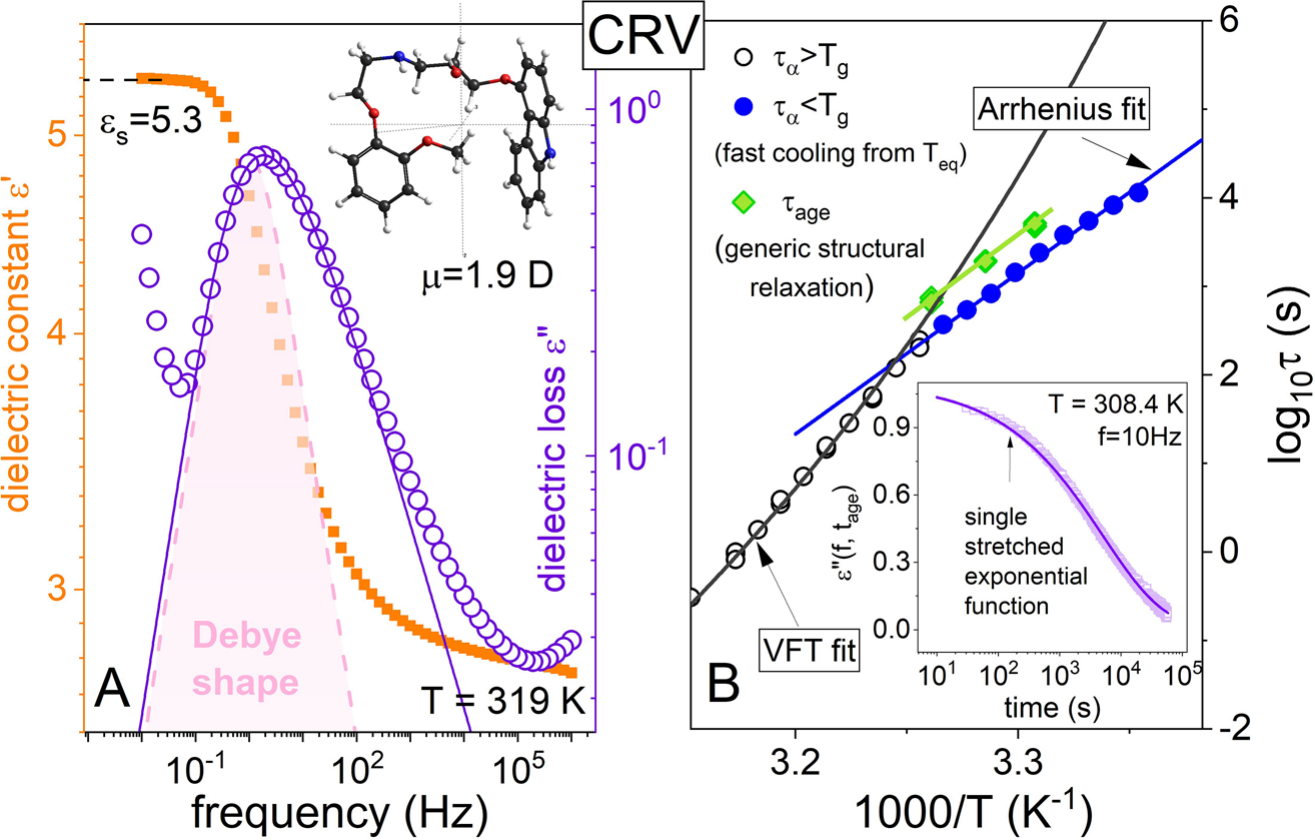
Dielectric data of CRV measured at 319 K (A). Solid line denotes the fit of the KWW function with β_KWW_ = 0.54. In the inset, the chemical structure of CRV is presented (red, oxygen; blue, nitrogen; black, carbon; gray, hydrogen). Panel B presents the temperature dependence of structural relaxation times of CRV determined above and below *T*_g_ together with the results from aging experiments. Inset: representative kinetic curve probing dielectric aging experiment at 302.3 K. A solid line is a fit of a single stretched exponential function (eq 1) to the experimental data with parameters τ_age_ = 4811 s and β_ag_ = 0.54. Parameters obtained at 304.3 K: τ_age_ = 1805 s and β_ag_ = 0.47; at 306.7 K: τ_age_ = 743 s and β_ag_ = 0.53.

Consequently, signs of cross-correlations are negligible in this material, and the structural relaxation peak observed in dielectric spectra reproduces mainly the generic structural relaxation. The representative result of the aging experiment, performed in the glassy state with the same protocol as it was for FPC, is depicted in the inset to Figure 3B. As expected, independent of *T*_age_, a single stretched exponential function is enough to parametrize the ε″(*f̃*, *t*_age_) data. The obtained values of the decay time τ_age_ versus inverse temperature are shown in Figure 3B and are listed in the figure caption together with β_ag_. At first sight, it can be noted that τ_age_ is longer than τ_α_ determined by using the TTS rule in the glassy state. However, only the point determined in the close vicinity of *T*_g_ (at 306.7 K) reaches the VFT curve. This provides additional confirmation of the Arrhenius behavior of structural dynamics in the glassy state.^24^

In summary, the dielectric experiments performed for van der Waals liquid FPC (classified as a chemical derivative of propylene carbonate) reveal strong polarity of this material reflected in high static dielectric constant ε_s_ equal to 180 in close vicinity of *T*_g_. At the same time, the narrow structural relaxation peak with β_KWW_ = 0.8 has been found for this compound. In turn, the isothermal aging experiments, performed at various *T*_age_ < *T*_g_, revealed the complex structural recovery dynamics of FPC. Specifically, a superposition of two stretched exponential functions was required to parametrize the ε″(*f̃*, *t*_age_) data precisely. Consequently, two characteristic aging time scales τ_age_ have been obtained at given *T*_age_. This result suggests a contribution of two different modes (cross-correlation and generic structural relaxation) to the aging dynamics of highly polar glass-formers. At the same time, similar experiments performed for weakly polar CRV (β_KWW_ = 0.54, ε_s_ = 5.3) provide a single value of τ_age_ at given *T*_age_, indicating that dipole–dipole interactions do not participate in the physical aging of CRV glass. These results support the recent ideas presented by the Blochowicz group on the substantial contribution of dipole–dipole interactions to the dielectric spectra of polar van der Waals liquids.^15^
